# Optineurin Shapes Basal and LPS-Induced Transcriptomes in BV2 Microglia

**DOI:** 10.3390/ijms262110453

**Published:** 2025-10-28

**Authors:** Sara Cappelli, Josip Peradinovic, Nikolina Mohovic, Purba Mandal, Cristiana Stuani, Alessandra Longo, Jason R. Cannon, Priyanka Baloni, Benedetta Leoni, Tamara Krsmanovic, Katica Stojanov, Gordana Apic, Robert B. Russell, Maurizio Romano, Emanuele Buratti, Ivana Munitic

**Affiliations:** 1 International Centre for Genetic Engineering and Biotechnology (ICGEB), Padriciano 99, 34149 Trieste, Italy; cristiana.stuani@icgeb.org (C.S.); alessandra.longo@icgeb.org (A.L.); emanuele.buratti@icgeb.org (E.B.); 2 Laboratory for Molecular Immunology, Faculty of Biotechnology and Drug Development, University of Rijeka, R. Matejčić 2, 51000 Rijeka, Croatia; josip.peradinovic@biotech.uniri.hr (J.P.); nikolina.mohovic@biotech.uniri.hr (N.M.); 3 School of Health Sciences, Purdue University, West Lafayette, IN 47907, USA; mandal11@purdue.edu (P.M.); cannonjr@purdue.edu (J.R.C.);; 4 Purdue Institute for Integrative Neuroscience, Purdue University, West Lafayette, IN 47907, USA; 5 Metisox—Cell Networks GmbH, 69120 Heidelberg, Germany; 6 BioQuant, Heidelberg University, 69120 Heidelberg, Germany; 7 Department of Life Sciences, University of Trieste, Via Licio Giorgieri 5, 34127 Trieste, Italy; mromano@units.it

**Keywords:** optineurin, RNA-Seq, BV2 microglia, LPS, inflammation, neuroinflammation, CRISPR-Cas9, interferon signaling

## Abstract

The *OPTN* gene, which encodes the adaptor protein optineurin, is genetically linked to amyotrophic lateral sclerosis and frontotemporal dementia, diseases characterized by chronic microglial activation. Optineurin regulates inflammatory signaling, autophagy, and trafficking, but its role in microglia remains incompletely understood. Here, we used bulk RNA sequencing to profile CRISPR-Cas9-mediated optineurin knockout (KO) and wild-type BV2 microglia under basal conditions and upon LPS stimulation. At baseline, optineurin KO altered ~7% of the transcriptome, with a predominant downregulation of type I interferon and antiviral pathways, suggesting its role in maintaining basal immune readiness. LPS stimulation reprogrammed ~35% of genes in wild-type microglia, inducing immune effectors and suppressing cell cycle regulators, whereas in optineurin-deficient cells, the response was blunted with only ~16% of genes changing relative to the KO baseline. Furthermore, LPS-treated optineurin KO microglia notably diverged from LPS-treated wild-type cells, with ~26% differentially expressed genes (DEGs). This included impaired induction of inflammatory programs and persistence of cell cycle-associated transcripts. Most DEGs in LPS-treated KO cells were unique to this condition, highlighting optineurin-dependent pathways specific to inflammatory challenge. Overall, our study provides a systems-level framework for investigating optineurin in microglia and neurodegeneration, establishing it as a key regulator of the microglial transcriptome, with its loss reshaping innate immune and cell cycle programs.

## 1. Introduction

Microglia are the resident immune cells of the central nervous system (CNS), critical for neural homeostasis and responses to injury or infection [1,2,3]. Chronic microglial activation is a hallmark of most adult-onset neurodegenerative diseases, including amyotrophic lateral sclerosis (ALS) and frontotemporal dementia (FTD) [2,4,5,6]. ALS and FTD form a disease spectrum due to substantial genetic and clinical overlap [7,8,9,10]. Optineurin, a ubiquitin-binding adaptor protein encoded by the *OPTN* gene, is of interest in neurodegeneration research due to its diverse roles in inflammatory signaling, autophagy, and vesicular/organelle trafficking, and because mutations in *OPTN* are linked to ALS, FTD, and both primary open-angle and normal-tension glaucoma [11,12,13,14,15]. Notably, pathogenic *OPTN* variants segregating in familial ALS include a nonsense mutation resulting in complete loss of optineurin and two distinct C-terminal mutations in the ubiquitin-binding domain—a missense mutation (E478G) and a truncation (Q398X) [12]. These mutations support loss-of-function as the likely mode of pathogenicity in ALS and FTD.

The specific mechanisms of disease-associated *OPTN* variants have remained difficult to decipher. Although optineurin is ubiquitously expressed, its functional consequences appear cell type- and stimulus-specific [16,17]. Conditional knockout studies in mice have revealed that optineurin loss in microglia or oligodendrocytes induced axonal degeneration, whereas deletion in neurons or astrocytes had no overt effect [18]. Notably, a recent study reported that C-terminal truncation of optineurin (Optn^470T^), when restricted to glutamatergic neurons, led to both normal-tension glaucoma and ALS-like symptoms in young adult mice [19]. By contrast, we have shown that whole-body Optn^470T^ mice did not develop overt neurodegeneration even at two years of age [20]. Similarly, several independently generated Optn^−/−^ mice showed no striking neurological phenotypes or shortened lifespan [18,21,22,23,24]. These divergent outcomes between whole-body and conditional models raise the possibility that optineurin in microglia has a protective role, potentially mitigating neuron-intrinsic defects driven by optineurin truncation. Understanding the transcriptional programs governed by optineurin in microglia is therefore important for understanding its role in CNS homeostasis and the pathogenesis of neurodegeneration.

Lipopolysaccharide (LPS) is widely used to model (neuro)inflammation in vivo and in vitro through Toll-like receptor 4 (TLR4) activation, which induces various innate immune responses, including pro-inflammatory cytokine and type I interferon (IFN) production [25,26,27,28]. The innate immune responses in the CNS, primarily orchestrated by microglia, have been implicated in the pathogenesis of neurodegenerative diseases, as pro-inflammatory mediators contribute to neuronal dysfunction and death [29,30]. On the other hand, microglia also exert numerous neuroprotective functions, highlighting their complex role in balancing tissue homeostasis and disease progression [5,31,32,33].

The role of optineurin in regulating microglial function remains unresolved due to conflicting phenotypes across Optn mouse models and reliance on in vitro studies largely restricted to selected functional readouts and targeted marker panels [18,19,20,21,22,23,24,34]. To address this gap, we systematically profiled optineurin-dependent transcriptional programs at baseline and during innate immune activation by combining CRISPR–Cas9-mediated optineurin deletion (Clustered Regularly Interspaced Short Palindromic Repeats: CRISPR-associated Protein 9) with LPS stimulation in BV2 microglial cells and performing bulk RNA sequencing (RNA-Seq) on four conditions: untreated wild-type (CTRL), optineurin knockout (KO), LPS-treated wild-type (LPS), and LPS-treated optineurin knockout (KO_LPS) cells. This work builds directly on prior studies showing optineurin as an important regulator of innate immune signaling in primary murine macrophages [21,35,36,37]. Extending these findings to microglia, we previously showed that optineurin is required for optimal TBK1/IRF3 activation and type I interferon responses following TLR stimulation in primary murine microglia [34]. We also demonstrated that optineurin KO in BV2 microglia phenocopies optineurin insufficiency in primary microglia from Optn^470T^ mice, with both models exhibiting TDP-43 accumulation and blunted responses to LPS stimulation [38]. This concordance supports the relevance of BV2 KO cells as a surrogate model for microglial optineurin loss-of-function in vivo. The present study employs systems-level transcriptomic profiling to characterize how optineurin deficiency in BV2 microglia shapes microglial responses under basal and inflammatory conditions. In the most informative contrast (KO_LPS vs. LPS), optineurin loss suppressed the induction of interferon/antiviral pathways, consistent with prior findings in inflammatory signaling in primary microglia [34], and additionally revealed retention of cell cycle programs, extending results from targeted assays to a much broader set of genes and pathways (4059 differentially expressed genes (DEGs) with concordant pathway enrichment). Such a framework offers insights into the role of optineurin in (neuro)inflammatory processes and generates hypotheses for future targeted validation.

## 2. Results

### 2.1. Optineurin Loss and LPS Drove Distinct Transcriptional Programs in BV2 Microglia

**Figure 1:**
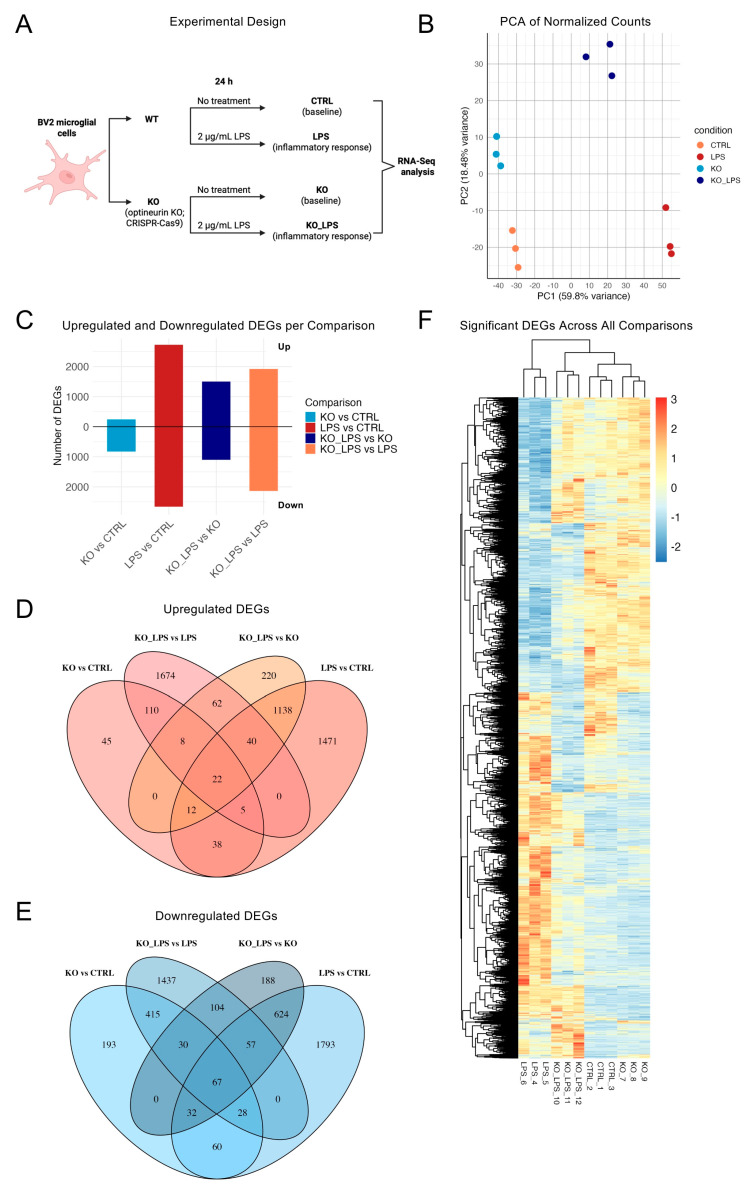
Optineurin loss and LPS induce distinct transcriptional programs in BV2 microglia. Differentially expressed genes (DEGs) were identified with DESeq2 (padj < 0.05, |log_2_FC| ≥ 0.5). (**A**) Schematic overview of the experimental design, showing the four conditions profiled by RNA-Seq: wild-type untreated control (CTRL), optineurin-knockout untreated (KO), wild-type + LPS (LPS), and optineurin-knockout + LPS (KO_LPS); created in BioRender. Munitić, I. (2025) https://BioRender.com/5l2stgi (accessed on 25 August 2025). (**B**) Principal component analysis (PCA) of variance-stabilized expression values from triplicate samples per condition. (**C**) Bar plot showing the number of significantly upregulated (positive *y*-axis) and downregulated (negative *y*-axis) DEGs for each pairwise comparison. (**D**,**E**) Venn diagrams show overlap of significantly upregulated (**D**, log_2_FC ≥ 0.5) and downregulated (**E**, log_2_FC ≤ –0.5) genes (padj < 0.05) across the indicated comparisons; directionality reflects labeled contrasts. (**F**) Unsupervised heatmap of the 7079 DEGs (padj < 0.05, |log_2_FC| ≥ 0.5) identified across all pairwise comparisons, with hierarchical clustering based on Euclidean distance of z-scored, variance-stabilized expression values.

To assess the impact of optineurin deletion on the BV2 microglia transcriptome at rest (baseline) and upon inflammatory challenge, we generated CRISPR/Cas9 optineurin KO BV2 cells. We previously showed that this approach led to a >90% decrease in optineurin protein levels in bulk cultures [38]. KO and the corresponding CTRL cells were either left untreated or were exposed to 2 µg/mL of LPS for 24 h (Figure 1A). Triplicate RNA-Seq libraries were prepared for each of the four conditions (CTRL, KO, LPS, KO_LPS). Principal component analysis (PCA) neatly separated samples along two orthogonal axes. PC1 (59.8% of the variance) segregated LPS-treated from untreated BV2 cells, and PC2 (18.48%) segregated KO from WT cells, with replicates within each group clustering tightly and underscoring technical reproducibility (Figure 1B). Notably, KO_LPS cells fell farthest from the other groups, indicating that the transcriptional impact of optineurin loss increased under inflammatory conditions. Combined, PC1 + PC2 explained ≈78% of total variance, confirming that LPS stimulation and optineurin status were the principal drivers of transcriptomic diversity. To test optineurin mRNA depletion, we examined its transcript levels (Supplementary Figure S1A). Optineurin mRNA levels in KO BV2 were reduced only by ~30%, contrasting >90% protein loss [38], potentially reflecting residual and/or altered transcripts or autoregulation. Optineurin mRNA increased by ~45% in LPS-treated WT cells and rose modestly in KO_LPS, consistent with its NF-κB inducibility [39]. Nonetheless, despite an incomplete depletion at the transcript level, optineurin KO exerted strong biological effects—as evidenced by distinct PCA separation, particularly under inflammatory conditions.

Differential expression analysis supported the PCA segregation data. The number of DEGs varied across conditions (Figure 1C). Among 15,450 quantified genes, the optineurin KO versus CTRL comparison identified 1065 significant DEGs—240 upregulated (1.6%) and 825 downregulated (5.3%)—demonstrating a notable but moderate role for optineurin in the basal state. In response to LPS, wild-type BV2 cells showed the strongest transcriptional shift (LPS vs. CTRL), with 5387 DEGs: 2726 up- and 2661 downregulated (17.6% and 17.2%, respectively; ~35% total). By contrast, KO_LPS versus (vs.) KO yielded 2518 total DEGs—1468 upregulated (9.5%) and 1050 downregulated (6.8%)—together representing ~16% of the transcriptome, indicating that optineurin KO cells underwent a substantially blunted transcriptional response to LPS. Notably, KO_LPS vs. KO showed more upregulated genes, indicating a loss of the usual LPS-driven repression seen in wild-type cells. Finally, KO_LPS versus LPS revealed 4059 DEGs (~26%)—1921 upregulated (12.4%) and 2138 downregulated (13.8%)—further underscoring the influence of optineurin on the inflammatory transcriptome. These global analyses indicated that while optineurin loss had a small but discernible effect at baseline, its impact was amplified during an inflammatory challenge, affecting both the number and distribution of DEGs.

Venn analysis revealed broad transcriptional divergence across comparisons (Figure 1D–E and Supplementary Figure S1B). Among upregulated genes, 1471 (54%) were uniquely induced in LPS-treated WT cells, while 1674 (87%) were unique to KO_LPS vs. LPS; similarly, 1793 (67%) downregulated genes were unique to LPS vs. CTRL and 1437 (67%) to KO_LPS vs. LPS, underscoring distinct optineurin-dependent responses to LPS. In contrast, only 45 (19%) and 220 (15%) upregulated genes were unique to KO vs. CTRL and KO_LPS vs. KO, respectively, and just 193 (23%) and 188 (17%) downregulated genes, showing that these contrasts were largely composed of shared signatures. A shared set of 110 upregulated genes (46% of KO vs. CTRL; 6% of KO_LPS vs. LPS) and 415 downregulated genes (50% of KO vs. CTRL; 19% of KO_LPS vs. LPS) overlapped between KO vs. CTRL and KO_LPS vs. LPS, highlighting some core optineurin-dependent programs that persisted under stimulation. Together, these patterns confirmed that optineurin modulated both basal and LPS-inducible transcriptional programs, with much stronger effects under stimulation.

To visualize the global structure of transcriptional variation across all conditions, we generated an unsupervised heatmap of the 7079 significant DEGs identified in pairwise comparisons (Figure 1F). Hierarchical clustering grouped samples into four well-separated branches: CTRL and KO formed proximal but distinct clusters, while LPS and KO_LPS samples branched further apart, with LPS exhibiting the greatest gene expression divergence from CTRL. This global pattern corroborated the PCA structure (Figure 1B), further confirming that the combination of optineurin loss and LPS stimulation induced a broad transcriptomic change. Moreover, the transcriptional distance from untreated CTRL samples was minimal for KO, pronounced for LPS, and reduced in KO_LPS samples relative to LPS. This suggested that optineurin loss attenuated the transcriptional response to LPS.

### 2.2. Optineurin Loss Reshaped the Basal BV2 Transcriptome

**Figure 2:**
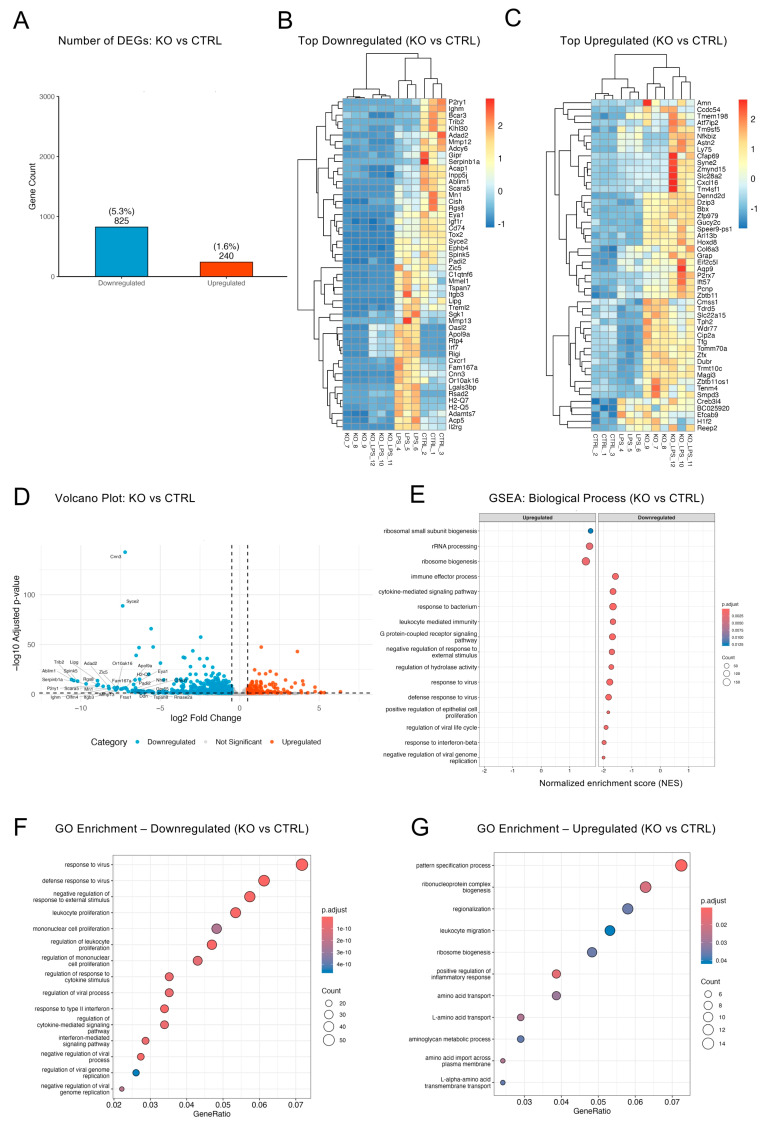
Optineurin sustained basal immune readiness while restraining growth-related transcriptional programs in BV2 microglia. (**A**) Bar plot showing the number and percentage of significantly up- and downregulated DEGs between KO and CTRL BV2 cells calculated using DESeq2 (padj < 0.05; log_2_FC ≥ 0.5). (**B**,**C**) Heatmaps of the top 50 most downregulated (**B**) and upregulated (**C**) genes in KO vs. CTRL, ranked by combined score (−log_10_[padj] × log_2_FC). Values are z-scored rlog-transformed counts; samples are clustered by Euclidean distance. (**D**) Volcano plot showing all DEGs in KO vs. CTRL ranked based on log_2_FC; top 50 DEGs are labeled. (**E**) GSEA bubble plot showing enriched GO Biological Processes based on pre-ranked log_2_FC values; *x*-axis = NES; color = padj; size = count; terms de-duplicated and capped at 16. (**F**,**G**) GO term enrichment analysis of significantly downregulated (**F**) and upregulated (**G**) genes. Dot size reflects GeneRatio; color indicates padj.

The finding that only ~7% of the quantified transcriptome was changed in optineurin KO versus CTRL BV2 cells, along with a marked skew toward downregulation, indicated that optineurin loss produced a targeted, rather than global, dysregulation in resting cells (Figure 2A and Figure 1C). To explore this selective transcriptomic shift, we first visualized the top down- and upregulated genes in KO versus CTRL BV2. Top downregulated DEGs had limited inter-sample variation, indicating a coordinated repression program (Figure 2B). Notably, in the top downregulated set, KO and KO_LPS clustered more closely with each other than with CTRL, suggesting a dominant effect of optineurin loss. The top upregulated DEG subset exhibited more heterogeneous expression and modest fold changes compared to CTRL, with greater variability among KO replicates (Figure 2C). Nevertheless, KO and KO_LPS samples still clustered more closely together than KO and CTRL. This imbalance between strong repression and moderate induction underscored the asymmetry of the basal optineurin signature. The volcano plot (Figure 2D) reinforced this asymmetry, with sharply downregulated genes extending below log_2_FC −10 and upregulated genes showing more modest fold changes, highlighting the skewed and selective impact of optineurin loss.

To compare the transcriptional makeup of KO to CTRL BV2 in more depth, we performed gene set enrichment analysis (GSEA). We observed a downregulation of interferon-, virus-, and immune-related pathways in KO cells (Figure 2E), which aligned with previous reports that have established optineurin as a positive regulator of the IRF3 pathway and type I interferon responses [34]. However, in contrast to prior reports describing effects after stimulation, our results suggested that optineurin also contributed to basal immune preparedness. In contrast, positive enrichment scores were dominated by biosynthetic pathways, including “rRNA processing” and “ribosome biogenesis”. Targeted GO Biological Process ontology overrepresentation analysis corroborated the GSEA findings. Downregulated terms (Figure 2F) included “response to virus”, “interferon-mediated signaling pathway”, and “cytokine-mediated signaling pathway”, whereas upregulated terms included “pattern specification process”, “ribosome assembly”, and several amino acid transport and metabolic pathways (Figure 2G). KEGG pathways analysis uncovered numerous repressed antiviral pathways in KO, including “herpesvirus infection” and “cytokine–cytokine receptor interaction” (Supplemental Figure S2A), whereas GO enrichment for upregulated genes was limited, reflecting small gene sets with modest changes (Supplemental Figure S2B). Consistent with these pathway-level patterns, several immune genes (*Il2rg*, *Cd74*, *H2-Q7*, *Lgals3bp*, *C1qtnf6*) appeared among the most strongly altered downregulated in KO vs. CTRL (Figure 2D). Furthermore, canonical interferon- and virus-stimulated genes (e.g., *Isg15*, *Ifit1/3*, *Oas2*, *Mx1*, *Stat1*, *Irf7*) were also significantly downregulated (Supplementary Table S1) but did not rank among the top-labeled genes, indicating that suppression of these pathways was broad yet not driven by strongly regulated genes. Together, these results indicated a functional rewiring of the resting BV2 transcriptome in optineurin KO cells, with immune defense programs suppressed and biosynthetic and metabolic processes modestly upregulated, supporting the view that optineurin sustained basal immune readiness while restraining growth-related pathways.

### 2.3. LPS Triggered a Broad Inflammatory Transcriptome in BV2 Cells

**Figure 3:**
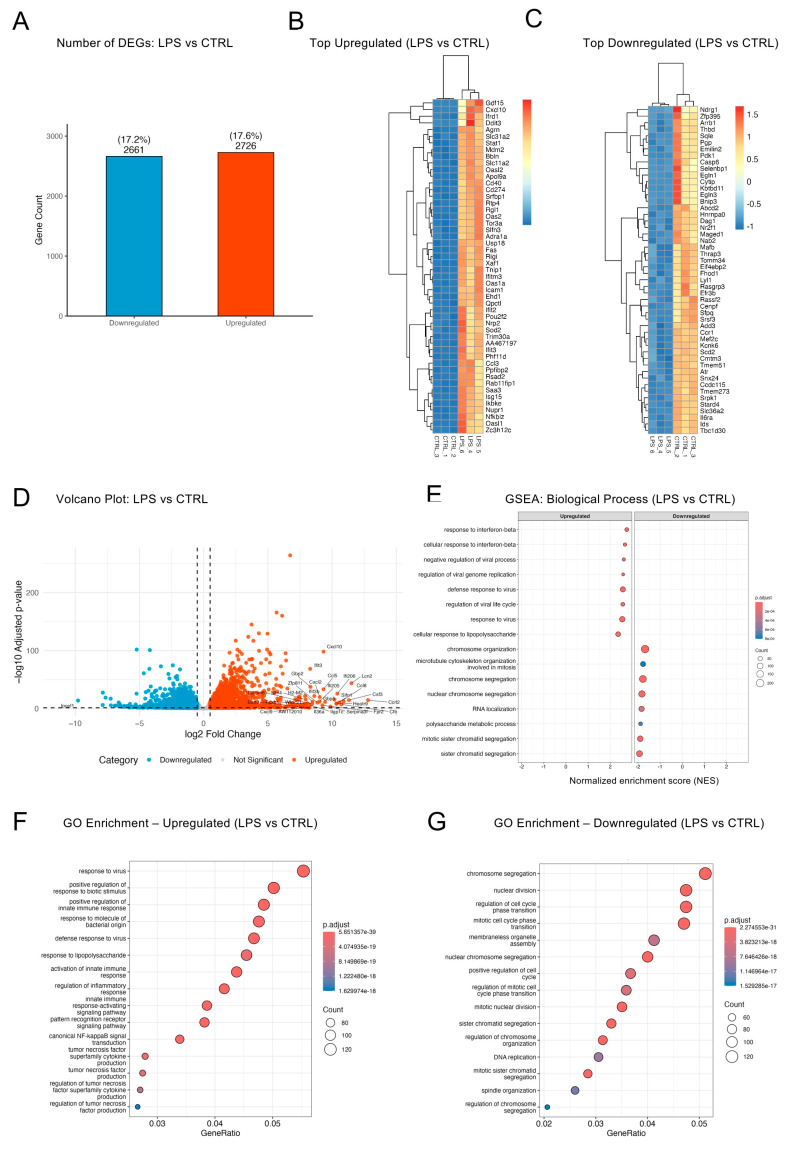
LPS reshaped a striking ~35% of the BV2 transcriptome. (**A**) Bar plot showing the number and percentage of DEGs in LPS vs. CTRL BV2 cells (padj < 0.05, |log_2_FC| ≥ 0.5). (**B**,**C**) Heatmaps of the top 50 most upregulated (**B**) and downregulated (**C**) genes in LPS vs. CTRL, ranked by combined score (−log_10_[padj] × |log_2_FC|). Rows represent genes; columns represent samples; values are z-scored variance-stabilized (rlog) counts. (**D**) Volcano plot shows significantly up- and downregulated DEGs ranked by log_2_FC, with top 50 DEGs labeled. (**E**) GSEA bubble plot (GO Biological Process) showing the top enriched terms in up- and downregulated genes. (**F**,**G**) GO overrepresentation dot plots; dot size = GeneRatio (query genes in term/term size). Only the top 15 enriched terms for upregulated (**F**) and downregulated (**G**) gene sets are displayed.

LPS stimulation strongly influences all myeloid cells, including microglia [40,41]. To test the effect of LPS in BV2 cells upon 24 h of stimulation, we compared LPS to CTRL. LPS reshaped a striking 35% of the transcriptome in BV2 cells, yielding 2726 upregulated and 2661 downregulated genes (Figure 3A and Figure 1C). To visualize high-magnitude DEGs across replicates, we plotted heatmaps of the most up- and downregulated transcripts (Figure 3B,C). LPS-treated samples clustered tightly and showed strong, uniform induction of top upregulated genes, while the repressed genes showed more modest fold changes and greater variability. These patterns highlighted a more robust and coordinated upregulation than repression in response to LPS.

The volcano plot illustrated both the breadth and amplitude of this bidirectional transcriptional response (Figure 3D). A markedly taller right-hand tail—extending beyond log_2_FC ≈ +11—indicated that upregulated genes exceeded the magnitude of repression, with top hits including chemokines (*Cxcl10*, *Ccl5*, *Cxcl2*), interferon-stimulated genes (ISGs) such as *Ifi205*, *Ifi206*, and *Ifit3b*, and innate immune effectors like *Csf3* and *Lcn2*. These genes encode canonical pro-inflammatory, antiviral, and cytokine pathway components, underscoring the potent activating effect of LPS. By contrast, the left-hand tail had fewer high-amplitude repressed genes, suggesting a less pronounced transcriptional repression. Top downregulated transcripts included *Gpr183* and *Ipcef1* (linked to immune homeostasis). Overall, this asymmetry in up- and downregulated profiles suggested that LPS exerted a stronger transcriptional push toward activation than repression (see Supplementary Table S2 for the full DEG list), even though the absolute numbers of upregulated and downregulated genes were balanced.

To determine how these gene-level changes translated into pathway shifts, we performed GSEA and GO overrepresentation analysis. The GSEA plot (Figure 3E) showed that leading-edge genes with positive enrichment scores were dominated by interferon and innate immune programs—including “response to interferon-beta”, “negative regulation of viral process”, and “defense response to virus”—whereas negative scores clustered in cell cycle and mitotic programs such as “chromosome organization/segregation”, “microtubule cytoskeleton organization involved in mitosis”, and “RNA localization”. GO enrichment of the upregulated genes reinforced these findings, highlighting “response to viruses”, “positive regulation of innate immune response”, “response to lipopolysaccharide”, and “canonical NF-κB signal transduction” (Figure 3F). In contrast, GO terms enriched among downregulated genes were dominated by cell cycle-related terms, including “chromosome segregation” and “nuclear division” (Figure 3G), suggesting that LPS diverts BV2 cells from proliferation toward an activated, anti-microbial state. Analysis of unique DEGs in the LPS vs. CTRL comparison additionally revealed enrichment of autophagy-related processes (Supplementary Figure S3). Together, these enrichment profiles showed that wild-type BV2 cells mounted a strong LPS-induced innate immune response while suppressing proliferation-related transcriptional programs.

### 2.4. Optineurin Deficiency Attenuated LPS-Induced Inflammatory Transcriptome

**Figure 4:**
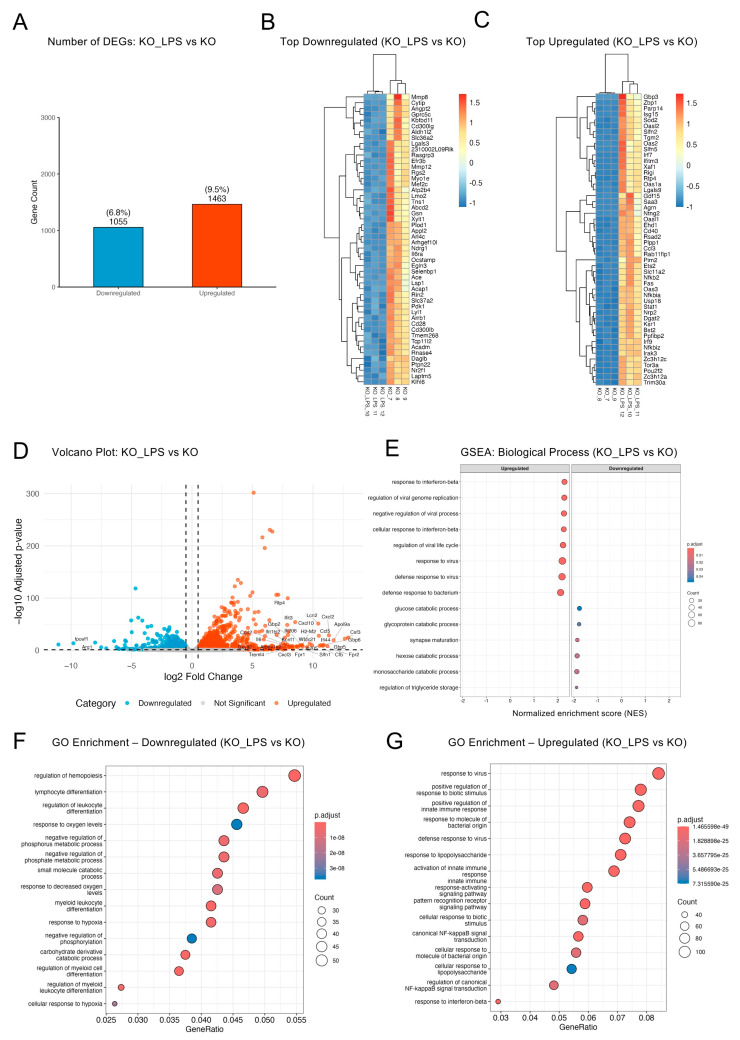
Optineurin loss reshaped the LPS-induced transcriptome by partially preserving immune activation but altering metabolic programs. (**A**) Bar plot showing significantly up- and downregulated genes in KO_LPS vs. KO (padj < 0.05; |log_2_FC| ≥ 0.5) using DESeq2. (**B**,**C**) Heatmaps of the top 50 downregulated (**B**) and upregulated (**C**) genes ranked by combined score (−log_10_[padj] × |log_2_FC|). Values are z-scored variance-stabilized (rlog) counts; samples and genes are hierarchically clustered. (**D**) Volcano plot shows significantly up- and downregulated DEGs ranked by log_2_FC, with top 50 DEGs labeled. (**E**) GSEA bubble plot (GO Biological Process) shows the top enriched pathways. (**F**,**G**) GO overrepresentation dot plots; dot size = GeneRatio (query genes in term/term size). Only the top 15 enriched terms for downregulated (**F**) and upregulated (**G**) gene sets are displayed.

Optineurin loss substantially altered the LPS-induced transcriptome, with 2518 DEGs in KO_LPS vs. KO (~16%; 1463 up; 1055 down; Figure 4A; Supplementary Table S3). Compared to the ~35% shift in LPS vs. CTRL (Figure 1C), this modest response indicated that optineurin loss attenuated LPS-induced programs. Heatmaps of top DEGs (Figure 4B,C) showed tight replicate clustering and coherent shifts relative to KO. The volcano plot (Figure 4D) highlighted strong induction of chemokines and inflammatory effectors (*Cxcl10*, *Ccl5*, *Cxcl2*, *Il1b*, *Il6*, *Csf3*, *Lcn2*) and ISGs (*Ifit3*, *Ifi205*), indicating preserved innate/antiviral activation. Fewer genes were repressed (e.g., *Arg1*, *Ipcef1*). These findings indicated that KO microglia retained the capacity to mount inflammatory and antiviral responses upon LPS challenge, but relative to WT LPS (Section 2.3), the response is narrower in scope and reduced in amplitude.

GSEA and GO overrepresentation found that many innate immune pathways enriched in wild-type LPS responses—including “response to interferon-beta” and “regulation of viral genome replication”—were also significantly enriched in KO_LPS versus KO cells (Figure 4E). However, unlike LPS, KO_LPS did not strongly repress mitotic and chromosome segregation programs. Instead, KO_LPS-specific downregulated pathways included several metabolic processes such as “glucose catabolic process”, “glycoprotein catabolic process”, and “regulation of triglyceride storage”, pointing to optineurin-dependent regulation of metabolic homeostasis. GO enrichment analyses reinforced these patterns (Figure 4F,G), with upregulated terms mirroring the innate immune profile of wild-type cells, including “response to virus”, “positive regulation of innate immune response”, and “canonical NF-κB signal transduction”; the downregulated set was uniquely enriched for lineage- and differentiation-related processes, including “regulation of hematopoiesis”, “lymphocyte differentiation”, and “response to oxygen levels”. Enrichment analysis performed only on the DEGs unique to KO_LPS vs. KO (220 upregulated and 188 downregulated; Figure 1D,E, Supplementary Figure S1B) revealed that the upregulated genes were enriched for cytoskeletal and motility-associated processes, whereas the downregulated genes were enriched for vesicular and lysosomal trafficking pathways (Supplementary Figure S4). This unique subset (~17% of DEGs) further underscored a qualitatively distinct transcriptional reprogramming in KO_LPS cells compared to wild-type responses. Altogether, these findings suggested that while canonical immune responses were partially preserved in the absence of optineurin upon LPS stimulation, they lacked the robust suppression of mitotic and chromosome segregation programs seen in wild-type cells and instead exhibited distinct metabolic shifts.

### 2.5. Optineurin Influenced Inflammatory and Cell Cycle Gene Regulation in Response to LPS

**Figure 5:**
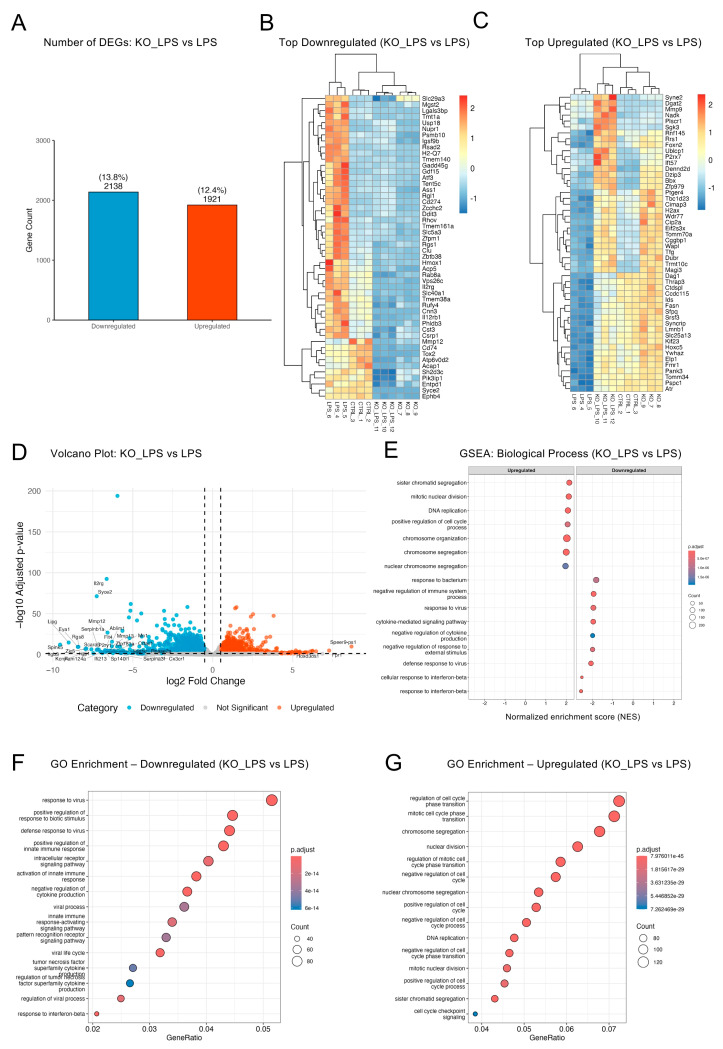
Optineurin influenced inflammatory and cell cycle gene regulation in response to LPS. (**A**) DEG counts for KO_LPS vs. LPS (padj < 0.05; |log_2_FC| ≥ 0.5); percentages and gene numbers are shown. (**B**,**C**) Heatmaps showing the top 50 most downregulated (**B**) and upregulated (**C**) genes; values are z-scored variance-stabilized (rlog) counts; samples and genes are hierarchically clustered. (**D**) Volcano plot shows significantly up- and downregulated DEGs ranked by log_2_FC, with top 50 DEGs labeled. (**E**) GSEA plot (GO Biological Process) highlights enriched pathways among up- and downregulated genes. (**F**,**G**) GO overrepresentation analyses of downregulated (**F**) and upregulated (**G**) gene sets. Only the top 15 enriched terms for downregulated (**F**) and upregulated (**G**) gene sets are displayed.

We next directly compared LPS-treated KO and WT cells (KO_LPS vs. LPS) to gain further insight into the role of optineurin in modulating the inflammatory transcriptome. This contrast yielded 4059 DEGs (~26% of the transcriptome; 2138 downregulated, 1921 upregulated; Figure 5A), consistent with the cumulative effects of both optineurin deficiency and differential LPS response. Heatmaps (Figure 5B,C) showed KO_LPS downregulated genes clustering with KO baseline and having uniformly lower expression compared to CTRL, indicating impaired inducibility; for upregulated genes, KO_LPS clustered with LPS but with variable amplitudes, consistent with partial activation. The partial overlap was further confirmed by analyzing the shared DEGs between LPS vs. CTRL and KO_LPS vs. KO pairs (1212 upregulated, 780 downregulated; Supplementary Figure S5A). Shared upregulated genes were dominated by innate immune- and pathogen-response pathways, including “response to bacterium”, “response to virus”, and “response to lipopolysaccharide”, confirming retention of a core LPS-driven program in KO microglia, whereas shared downregulated genes highlighted suppression of metabolic and stress-adaptive processes such as “glucose catabolic process” and “response to decreased oxygen levels”. In contrast, the transcriptional divergence between KO_LPS and LPS was clearly visible in the volcano plot (Figure 5D). The left-hand tail featured a substantially larger number of significantly downregulated genes compared to the right-hand tail, confirming that optineurin loss primarily resulted in failed induction of transcripts normally upregulated by LPS. Among the most strongly repressed genes were *Cx3cr1*, *Rnase2a*, *Lipg*, *Zic5*, *Rgs8*, *IL2rg*, *P2ry1*, *Mmp12* and *Mmp13*—a set that includes regulators of lipid metabolism, signal transduction, and immune modulation. In contrast, the top upregulated genes in KO_LPS compared to LPS—such as *Speer9-ps1* and *Hoxd3os1*—were fewer and exhibited more modest fold changes. This pattern, also evident in Supplementary Table S4, indicated reduced LPS responsiveness in optineurin-deficient BV2 cells.

GSEA on the KO_LPS vs. LPS comparison revealed that downregulated genes were enriched for innate immune and antiviral pathways (Figure 5E), including “response to interferon-beta” and “defense response to virus”. These results suggested that many LPS-inducible immune programs failed to properly activate in optineurin-deficient cells. In contrast, the upregulated genes showed enrichment for cell cycle-related processes such as “sister chromatid segregation”, “mitotic nuclear division”, and “DNA replication”, indicating that KO_LPS cells retained proliferative signatures that were suppressed by LPS in WT cells. GO analysis of downregulated genes (Figure 5F) highlighted immune processes (“response to virus”, “positive regulation of innate immune response”), while analysis of upregulated genes (Figure 5G) highlighted cell cycle and chromosomal programs (“cell cycle phase transition”, “chromosome segregation”, “nuclear division”). Notably, several of these pathways were uniquely enriched in the KO_LPS vs. LPS comparison and not observed in other contrasts. Specifically, DEGs unique to KO_LPS vs. LPS (1674 upregulated, 1437 downregulated; Figure 1D,E, Supplementary Figure S1B showed strong enrichment of mitotic/chromosomal programs among the upregulated set and innate immune/interferon beta-related terms among the downregulated set (Supplementary Figure S5B). Reactome enrichment analysis (Supplementary Figure S5C) reinforced these findings, with persistent activation of mitotic checkpoints and M-phase programs. KEGG pathway analysis (Supplementary Figure S5D,E) identified downregulation of viral infection pathways (e.g., Epstein–Barr virus, herpes simplex virus, influenza A) and upregulation of cell cycle and senescence pathways. Interestingly, some bacterial and viral KEGG pathways, such as “*Salmonella* infection” and “HTLV-I infection,” were selectively enriched in the upregulated gene set, underscoring a complex and non-uniform transcriptional landscape in optineurin-deficient cells. Taken together, these analyses suggested that optineurin loss weakened immune activation while preventing the cell cycle suppression linked to LPS stimulation. Thus, the key finding here is not only a general dampening of inflammation but also a rewiring of the microglial program: KO_LPS cells failed to suppress cell cycle genes upon LPS, implicating optineurin in the transition from a proliferative to an immune effector state.

**Figure 6:**
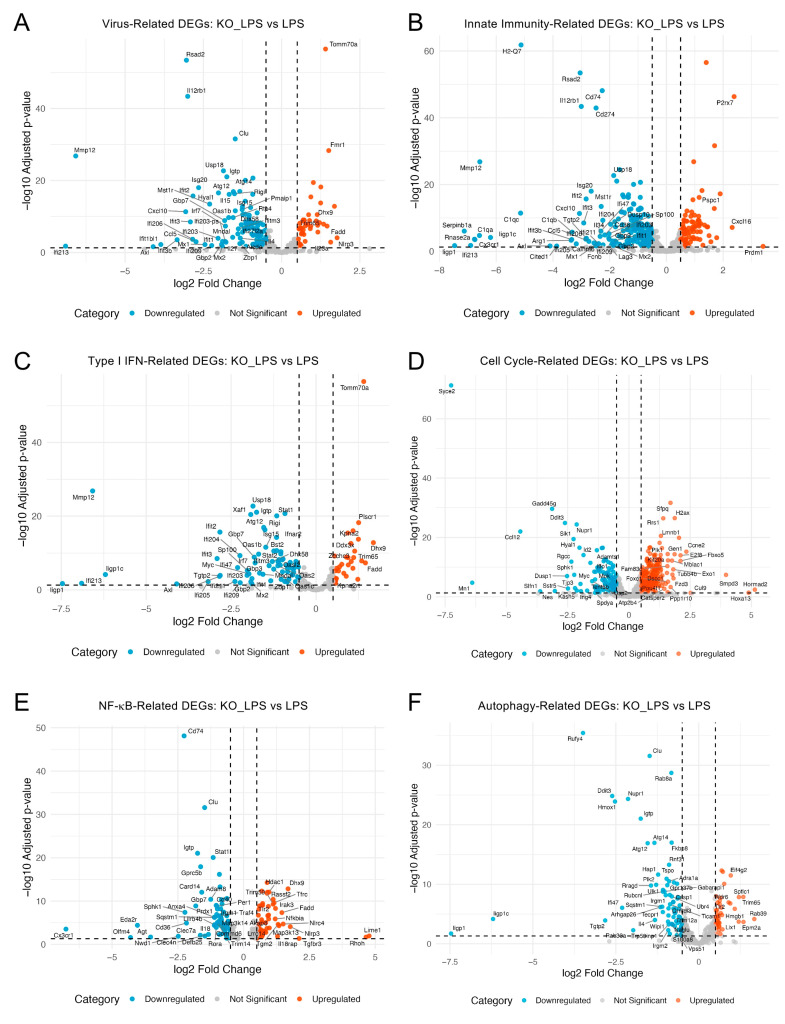
Targeted transcriptional profiling of functional gene sets in KO_LPS vs. LPS. (**A**–**F**) Volcano plots display differentially expressed genes from selected functional categories enriched in the KO_LPS vs. LPS comparison. Virus- (**A**), innate immunity- (**B**), type I interferon- (**C**), cell cycle- (**D**), NF-κB- (**E**), and autophagy-related (**F**) DEGs are shown. Gene sets were defined based on GO BP terms. Top 50 DEGs (padj < 0.05), ranked by log2fc are labeled.

To further characterize the transcriptional effects of optineurin loss under inflammatory conditions, we analyzed individual genes from key biological programs enriched in the KO_LPS vs. LPS contrast (including viral defense, innate immunity, type I interferon, and cell cycle), as well as NF-κB and autophagy pathways previously linked to optineurin function [14,42]. Volcano plots (Figure 6A–F) showed broad downregulation of virus- (A), innate immunity- (B), and type I interferon-related genes (C) in KO_LPS samples, including genes like *Irf7*, *Isg15*, *Stat1*, *Cxcl10*, *Cxcl5*, *Cx3cr1*, *Rsad2*, *Usp18*, *Mmp 12*, *C1qa* and *Ddx58*. A few transcripts (*Tomm70a*, *Nlrp3*, *Cxcl16*, and *Plscr1*) were modestly upregulated, but antiviral and inflammatory genes were broadly suppressed. The cell cycle-related genes (Figure 6D) were skewed toward upregulation, with numerous mitotic markers increased, alongside a minority that were decreased. NF-κB- and autophagy-related genes (Figure 6E) showed a mixed response, suggesting that the optineurin loss changes are selective rather than global.

Finally, we analyzed differentially expressed transcription factors (TFs) across key comparisons (Supplementary Figure S6). In both LPS vs. CTRL and KO_LPS vs. KO, mRNAs for canonical inflammatory TFs such as Stat1, Stat2, Irf1, Irf7, and Relb were strongly induced, consistent with activation of interferon and NF-κB signaling (Supplementary Figure S6A). However, in the KO_LPS vs. LPS comparison, many TFs, in particular Irf7, were significantly downregulated, suggesting a blunted or dysregulated LPS response in optineurin KO cells. In contrast, mRNAs for developmental/lineage specification TFs (homeobox: Hoxa13, Hoxc5; forkhead: Foxc1; bZIP: Atf3, Cebpd) changed specifically in KO_LPS vs. LPS, but not in the other contrasts, suggesting compensatory or maladaptive activation. Heatmaps supported these findings and revealed clustering of samples according to treatment, with immune response-related TFs elevated in LPS but suppressed in KO_LPS, and developmental TFs enriched in KO_LPS (Supplementary Figure S6B). Taken together, these results suggested that optineurin loss decoupled LPS-driven inflammatory TF programs and engaged alternative TF networks.

### 2.6. Targeted Literature Mining for Potential Modulators of Optineurin-Mediated Pathways

Because optineurin functions as a TBK1 adaptor controlling IRF3 activation [14,43], and optineurin loss blunted virus-, innate immunity-, and type I interferon-related genes in KO_LPS samples (Figure 6A–C), we searched for the modulators of these dysregulated pathways. Given that optineurin and TBK1 have been reported to have both neuroprotective and neurotoxic functions in different models [18,19,44], we searched for both activators and inhibitors in the IUPHAR/BPS Guide to PHARMACOLOGY database. For downregulated genes, we identified small-molecule inhibitors for CX3CR1, MMP12, CXCL5 and CXCL10, and an oligonucleotide agonist for RIG-I, whereas for upregulated genes, we identified small-molecule inhibitors for NLRP3 (Supplementary Figure S7). For TBK1, we identified the approved drug amlexanox and additional small-molecule inhibitors. The proteins for which IUPHAR does not report any modulators are IRF3, optineurin, IRF7, ISG15, STAT1, RSAD2, USP18, C1QA, TOMM70 (TOMM70A), and PLSCR1. Thus, targeted IUPHAR/BPS queries mapped dysregulated targets to corresponding modulators, providing a hypothesis-generating basis for future validations.

## 3. Discussion

The role of optineurin in ALS and FTD has been difficult to decipher due to its pleiotropic functions and pronounced cell-type specificity [14,16,17,42]. While its involvement in inflammatory signaling and autophagy is well recognized [35,36,45,46,47,48], its direct impact on microglia remains poorly characterized, as most previous studies focused on non-microglial systems or lacked genome-wide resolution. This gap is further underscored by contrasting findings from in vivo models: a recent study showed that C-terminal truncation of optineurin in glutamatergic neurons caused early-onset neurodegeneration in mice [19], whereas whole-body optineurin truncation in our hands produced no overt phenotype and no pathological signs of neuroinflammation or neurodegeneration, even in two-year-old animals [20]. Notably, both studies were based on the identical C-terminally floxed optineurin allele (Optn^470T^) originally generated by us [36], arguing that the divergent outcomes do not stem from differences in the optineurin variant used. These divergent outcomes raise the possibility that microglia may even play a compensatory or protective role in optineurin-related pathogenesis. Earlier work on this topic, including our own, addressed the immunoregulatory roles of optineurin in primary murine myeloid cells but employed classical molecular and immunological methods without using transcriptome-wide approaches [21,34,35,36,49,50]. To address critical knowledge gaps in optineurin-mediated functions in microglia, we applied a systems-level RNA-Seq approach to comprehensively characterize optineurin-dependent transcriptional programs under basal and inflammatory conditions in BV2 microglia.

Here we found that optineurin loss had a modest but selective impact at baseline, altering ~7% of the BV2 transcriptome, with a marked skew toward gene downregulation. A significant magnitude of change for a modest percentage of genes affected suggests selective targeting of biochemical pathway(s). Downregulated genes in optineurin KO BV2 were enriched for innate immune functions. These findings are consistent with prior work indicating that optineurin regulates inflammatory signaling downstream of pattern recognition receptors [21,34,35,36]. Moreover, the findings here showed that several of the immune functions are affected even in the absence of apparent stimulation, which, to our knowledge, has not been reported previously. In contrast, upregulated transcripts were dominated by biosynthetic and metabolic pathways that have not been previously linked to optineurin function. One recent study linked optineurin to NRF2 antioxidant signaling [51], suggesting a broader role of optineurin in cellular homeostasis. The data reported here add to this view by showing that optineurin supported basal immune readiness in resting microglia while actively constraining some biosynthetic and metabolic programs. This is potentially linked to previous findings that showed that optineurin regulates interferon responses in a cell cycle-dependent manner [52], providing a possible link between the upregulation of biosynthetic pathways with suppressed immune gene expression in optineurin-deficient cells. Collectively, these findings expand the known scope of optineurin function at steady state.

Under inflammatory stimulation, the effects of optineurin loss became markedly larger in scale. Compared to wild-type BV2 cells, which exhibited a broad transcriptional shift affecting ~35% of genes, LPS-treated KO cells showed a significantly reduced response, with only ~16% of genes differentially expressed relative to their unstimulated baseline. This finding indicated that optineurin deficiency compromised the BV2 microglial ability to adequately respond to an immune challenge. TLR4 stimulation via LPS activates two distinct signaling pathways, TBK1/IRF3 and NF-κB [25]. The activation of the former results in IFN-β production, which stimulates the interferon α/β receptor (IFNAR) and promotes further production of the IFN-stimulated genes (ISGs) such as *Irf7* [53]. In this study, we were unable to detect induction of IFN-β mRNA itself, likely due to the late 24 h timepoint, since IFN-β mRNA peaks within the first hours of stimulation and drops by 24 h [34,54]. Nevertheless, IFN-β-mediated signaling was evident by increased expression of numerous ISGs, such as *Irf7*, *Stat1*, and *Isg15* in LPS-treated WT cells, all of which were significantly decreased in KO_LPS samples. Thus, KO_LPS samples failed to robustly induce and sustain expression of key antiviral and IFN-stimulated genes, consistent with reduced enrichment of immune-related pathways. Notably, activation of NF-κB and autophagy pathway genes was more heterogeneous. Unique LPS-WT DEGs showed autophagy term enrichment, but in KO_LPS both NF-κB- and autophagy-related gene sets displayed mixed up- and downregulated genes. Given that these pathways are heavily regulated post-transcriptionally, additional proteomic studies are necessary. Our previous findings in LPS-stimulated Optn^470T^ primary neonatal microglia showed dysregulation of numerous IFN-β targets, but no major effects on the NF-κB pathway [34]. Additionally, similar results were reported in other optineurin-deficient and insufficient primary myeloid cells upon LPS stimulation [21,35,36]. The incomplete activation of canonical antiviral effectors, together with insufficient induction of *Irf7*, *Isg15*, *Ifit206*, *Oas1b* and *Usp18* (critical for feedback control of IFN type I signaling), suggests that optineurin is required not only for pathway initiation but also for full transcriptional execution of antiviral programs. Moreover, direct comparison of LPS-treated KO and WT BV2 cells revealed over 4000 differentially expressed genes, the majority of which were downregulated in KO_LPS, indicating that optineurin is required for full transcriptional engagement of LPS-inducible programs. Importantly, 87% of upregulated and 67% of downregulated DEGs in KO_LPS were unique to this condition, further underscoring the context-specific regulatory role of optineurin during inflammatory activation. At the same time, KO_LPS cells retained or even upregulated expression of mitotic and biosynthetic pathways that were suppressed in wild-type cells, suggesting the impact of optineurin on cell cycle upon inflammatory challenge. This is consistent with GO term enrichment showing increased expression of genes involved in “chromosome segregation,” “DNA replication,” and “mitotic nuclear division” in KO_LPS, pointing toward defective transition into the non-proliferative, immune-activated state typical of LPS-exposed microglia. Since LPS was previously reported to exert a cell cycle arrest in BV2 [55], the finding that KO_LPS retained cell cycle and biosynthetic programs indicated that optineurin is required to impose this proliferative brake during inflammatory activation. This suggests that optineurin loss dysregulated LPS-mediated activation and metabolic reprogramming by uncoupling biosynthetic from immune functions. An uncoupling has also been described in primary disease-associated microglia (DAM) in AD and ALS models, which downregulate homeostatic while upregulating lipid/lysosomal and phagocytic markers, reinforcing that activation can proceed with separation from homeostatic functions [56]. Together, these findings suggested that optineurin coordinates both activation and resolution arms of the LPS response, coupling immune induction with cell cycle arrest to promote an appropriate microglial activation state.

The blunted transcriptional response to LPS in OPTN-KO BV2 cells aligns with reports from a related TBK1 haploinsufficiency ALS model (Tbk1^+/−^), which perturbs the TBK1–optineurin axis [44]. Tbk1^+/−^ microglia showed dampened LPS-induced innate programs with downregulation of mRNAs for many ISGs mapped in our study, such as *Irf7*, *Irf9*, *Stat1*, *Stat2* and *Isg15*. Notably, TBK1 haploinsufficiency in the superoxide dismutase 1 (SOD1) G93A mouse model precipitated earlier disease onset yet prolonged lifespan, demonstrating the potential protective effect of diminished TBK1–optineurin axis at the late disease stages. Together with our KO_LPS data—where innate immune programs were blunted—these findings support a dual role of microglial activation: reduced TBK1–optineurin signaling may compromise acute immune responses, yet later in disease, it may mitigate chronically harmful neuroinflammation. This potentially protective role of optineurin deficiency and/or insufficiency may help explain the discrepancy between the Optn^470T^ glutamatergic neuron-restricted model, which develops neurodegeneration [19], and the whole-body Optn^470T^ model, which remains overtly normal [20], if microglial Optn^470T^ exerts a neuroprotective effect by dampening excessive inflammation. However, the protective role is likely context-specific and needs further study, as perturbation of optineurin or TBK1 can also result in toxicity. For example, mice carrying the human TBK1 p.E696K variant that specifically abrogates optineurin binding developed autophagolysosomal defects, which precipitated the age-associated motor neuron disease [57]. Similarly, selective microglial TBK1 loss drove proinflammatory and aging microglial signatures [58]. It would be important to test if chemical modulation of the TBK1–optineurin axis, especially the dysregulated immune-related proteins identified here, may affect ALS disease progression. In the IUPHAR/BPS Guide to PHARMACOLOGY database, we found several potential modulators of the TBK1–optineurin axis, including the TBK1 inhibitor amlexanox, a drug with prior clinical use in atopic conditions and potential therapeutic applications in metabolic and inflammatory diseases [59]. Indeed, amlexanox was recently prioritized as a potential drug repurposing target for ALS [60]. Considering a possible protective role of optineurin deficiency, inhibiting the TBK1–optineurin axis may benefit disease contexts or stages with TBK1 hyperactivity. However, because TBK1 loss-of-function mutations cause familial ALS/FTD [44,61,62], use of such modulators should be guided by patient genotype and disease stage and supported by validation in cellular and animal models.

While our study provides a comprehensive transcriptomic analysis of optineurin function in microglia, limitations should be noted. The analyses were conducted in the BV2 microglial cell line, which, despite its utility and reproducibility, differs from primary microglia. Furthermore, transcript-level changes do not always correlate with protein abundance or function, as shown in other microglial studies [63]. However, this study builds directly upon our prior mechanistic work in which we validated optineurin-dependent modulation of key signaling pathways, including TBK1/IRF3, and its impact on TDP-43 protein levels [34,36,37,38,49]. Accordingly, we consider the BV2 RNA-Seq results hypothesis-generating and will prioritize validation of the key inflammatory, cell cycle, and metabolic targets identified here via functional assays in relevant ALS/FTD models.

## 4. Materials and Methods

### 4.1. Generation of Optineurin Knockout Cells, Cell Culture and Treatments

The BV2 microglial cell line (a kind gift from Dr. J. Kriz; originally from ATCC) was maintained in Dulbecco’s Modified Eagle Medium (DMEM) supplemented with 10% fetal bovine serum (FBS), 2 mM L-glutamine, and antibiotic/antimycotic solution (10,000 U/mL penicillin, 10 mg/mL streptomycin, 25 µg/mL amphotericin B), hereafter referred to as complete DMEM. We previously generated optineurin knockout (KO) microglial BV2 cells by the CRISPR/Cas9 approach, targeting the third exon of the *Optn* gene with two gRNAs [38]. KO cells were selected with 2 μg/mL of puromycin (Carl Roth, Karlsruhe, Germany; Cat. No. 0240.2), based on the lowest concentration that resulted in 100% cell death in non-transfected controls. KO BV2 cells were maintained in complete DMEM with puromycin until seeding for the experiment. For this study, cells were seeded at 5 × 10^5^ cells per well in 6-well plates. After 24 h, CTRL and KO BV2 cells were either left untreated or stimulated with 2 µg/mL lipopolysaccharide (6000 EU/mL) (LPS; Sigma-Aldrich via Merck, Darmstadt, Germany; Cat. No. L4391) for an additional 24 h. Cells were then harvested for total RNA isolation for transcriptomic analysis. All four experimental conditions (CTRL, KO, LPS, KO_LPS) were prepared in biological triplicates, i.e., from three independent experiments.

### 4.2. RNA Isolation, Sequencing, and Transcriptomic Analysis

Total RNA was isolated using the RNeasy Mini Kit (QIAGEN, Venlo, Netherlands; Cat. No. 74104) according to the manufacturer’s instructions. RNA concentration and purity were assessed on a BioDrop Duo spectrophotometer; all samples had 260/280 and 260/230 ratios of ~2 and were deemed suitable for sequencing. mRNA library preparation (poly-A enrichment) and RNA sequencing were performed by Novogene (https://en.novogene.com/, accessed on 17 November 2024) on biological replicates from three independent experiments, as described above. RNA-seq analysis was performed using an Illumina NovaSeq 6000 instrument (Illumina, San Diego, CA, USA), generating 150 bp paired-end reads (PE150), with ~94–113 million read pairs per sample. Read quality was high, with ≥99.9% of bases achieving Q30 scores. The original raw data from Illumina were transformed into sequenced reads by CASAVA base recognition. Low-quality reads (>50% reads with nucleotide quality value ≤ 5 or >10% reads with uncertain nucleotides) or reads containing adapters were removed from the analysis. Clean reads were mapped to the reference Mus musculus genome (GRCm38.p6) using Hisat2 (v2.0.5), and featureCounts (v1.5.0-p3) was used to count the number of reads mapped to each gene [64,65]. Transcript assembly and novel transcript prediction were performed on the mapped reads of each sample assembled by StringTie (v1.3.3b) [66]. GSEA and overrepresentation analysis of Gene Ontology (GO) terms and Kyoto Encyclopedia of Genes and Genomes (KEGG) pathways were performed in RStudio (v2025.05.0.496) using R (v4.5.1). In particular, differential gene expression analysis was carried out using the DEseq2 package (v1.40.0) [67]. For overrepresentation analyses, genes were considered significantly differentially expressed if they had a Benjamini–Hochberg false discovery rate (FDR)-adjusted *p*-value (padj) < 0.05 and an absolute log_2_ fold change (|log_2_FC|) ≥ 0.5 [68]. Volcano plots, bar plots, heatmaps, and Venn diagrams were generated using the ggplot2 package (v3.5.2). Heatmaps were generated from variance-stabilized counts (DESeq2 vst), z-scored per gene and clustered unsupervised (rows and columns) by hierarchical clustering with Euclidean distance and complete linkage using R pheatmap. GSEA dotplots ranked terms by normalized enrichment score (NES); overrepresentation dotplots ranked by GeneRatio. Only curated gene symbols were labeled in the volcano plots and heatmaps (e.g., excluding Gm, LOC, Rik, ENS); volcano labels = top DEGs by |log_2_FC|, and heatmaps were ranked by −log_10_(padj) × |log_2_FC|. The ClusterProfiler package (v4.16.0) was used for overrepresentation analyses using GO terms and KEGG pathways, as well as GSEA of RNA-Seq data [69]. GO Biological Process (BP) terms were used to identify significantly affected transcriptional signatures. For GO overrepresentation analysis, DEGs were filtered by adjusted *p*-value < 0.05 and GeneRatio > 0.02. To assess shifts within specific biological processes, we used either keyword-filtered or manually curated GO BP terms. Genes associated with viral response, autophagy, innate immunity, and cell cycle regulation were extracted using broad GO term filters for the phrases “virus”, “autophagy”, “innate immune”, or “cell cycle”. Curated term sets were used for type I IFN and NF-κB. Specifically, we used 25 GO terms for IFN type I (including GO:0034340, response to type I interferon; GO:0035456, response to interferon-beta; and GO:0060340, positive regulation of type I interferon-mediated signaling pathway) and 17 for NF-κB (including GO:0007252, I-kappaB phosphorylation and GO:0043122, regulation of canonical NF-kappaB signaling); the full list is provided in Appendix A. In parallel with GO-based analysis, we also performed pathway enrichment using KEGG and Reactome databases. KEGG provides curated metabolic and signaling pathways, while Reactome offers expert-curated molecular interaction networks.

### 4.3. Literature-Based Identification of Potential Modulators

To identify potential chemical modulators that may target optineurin-mediated pathways, we searched for modulators of the TBK1–optineurin axis and of the selected dysregulated immune-related proteins from the KO_LPS vs. LPS comparison. We selected relevant DEGs from virus-, innate immunity-, and type I interferon-related pathways (*C1qa*, *Cx3cr1*, *Cxcl10*, *Cxcl5*, *Irf7*, *Isg15*, *Mmp12*, *Plscr1*, *Ddx58 (RIG-I)*, *Rsad2*, *Stat1*, *Usp18*; *Tomm70a*, *Nlrp3*, *Cxcl16*). We used the IUPHAR/BPS Guide to PHARMACOLOGY as a tool to identify approved drugs, inhibitors, and activators [70]. We used the SysWiz editor (version 3.0.4) for schematic representation [71]. SysWiz is a tool similar to ToxWiz [72], only augmented with curated information on ciliary diseases from the FP7 European Project Syscilia.org about human-validated relationships and by literature-derived relationships with human genes/proteins.

### 4.4. Data Availability

The raw and processed RNA-sequencing data generated for this study have been deposited in the NCBI Gene Expression Omnibus (GEO) database. The data are accessible through the GEO Series accession number GSE307044.

### 4.5. Use of Generative AI

ChatGPT (OpenAI, San Francisco, CA, USA; versions GPT-4 and GPT-5) was used to assist with language editing and code assistance. All scientific content and interpretations were provided by the authors.

## 5. Conclusions

Our findings identified optineurin as a critical regulator of microglial immune programs under both homeostatic and inflammatory conditions. Optineurin loss resulted in broad transcriptomic alterations, affecting especially type I interferon, innate immune and cell cycle pathways. These insights contribute to the understanding of microglial biology and may have relevance for neurodegenerative diseases in which optineurin and microglial dysfunction are implicated.

## Data Availability

The original contributions presented in this study are included in the article/Supplementary Materials. Further inquiries can be directed to the corresponding authors.

## Supplementary Materials

The following supporting information can be downloaded at https://www.mdpi.com/article/10.3390/ijms262110453/s1.

## Abbreviations

The following abbreviations are used in this manuscript:

ALS	Amyotrophic Lateral Sclerosis
ATCC	American Type Culture Collection
BV2	BV2 Microglial Cell Line
CNS	Central Nervous System
Cas9	CRISPR-associated Protein 9
CRISPR	Clustered Regularly Interspaced Short Palindromic Repeats
CTRL	Control (Wild Type, Untreated)
DEG	Differentially Expressed Gene
DESeq2	Differential Expression analysis using Sequencing, version 2
DMEM	Dulbecco’s Modified Eagle Medium
FBS	Fetal Bovine Serum
FTD	Frontotemporal Dementia
GEO	Gene Expression Omnibus
GO	Gene Ontology
GSEA	Gene Set Enrichment Analysis
IFN	Interferon
IFNAR	Interferon α/β receptor
IRF	Interferon Regulatory Factor
ISG	Interferon-Stimulated Gene
KEGG	Kyoto Encyclopedia of Genes and Genomes
KO	Knockout
KO_LPS	Optineurin Knockout + LPS
LPS	Lipopolysaccharide
NFκB	Nuclear Factor Kappa Light Chain Enhancer of Activated B Cells
NRF2	Nuclear factor erythroid 2-related factor 2
PCA	Principal Component Analysis
RNA-Seq	RNA Sequencing
TBK1	TANK binding kinase 1
TF	Transcription Factor (used in Section 2.5 and Section 2.6).
TLR4	Toll-Like Receptor 4
WT	Wild Type

## Appendix A

### Curated GO Terms for Targeted Pathway Analysis

Type I Interferon GO Terms Used for Targeted Gene Set Extraction:

GO:0004905 (type I interferon receptor activity), GO:0005132 (type I interferon receptor binding), GO:0019962 (type I interferon binding), GO:0032479 (regulation of type I interferon production), GO:0032480 (negative regulation of type I interferon production), GO:0032481 (positive regulation of type I interferon production), GO:0032606 (type I interferon production), GO:0032607 (interferon-alpha production), GO:0032608 (interferon-beta production), GO:0032647 (regulation of interferon-alpha production), GO:0032648 (regulation of interferon-beta production), GO:0032687 (negative regulation of interferon-alpha production), GO:0032688 (negative regulation of interferon-beta production), GO:0032727 (positive regulation of interferon-alpha production), GO:0032728 (positive regulation of interferon-beta production), GO:0034340 (response to type I interferon), GO:0035456 (response to interferon-beta), GO:0035457 (cellular response to interferon-alpha), GO:0035458 (cellular response to interferon-beta), GO:0038197 (type I interferon receptor complex), GO:0039502 (symbiont-mediated suppression of host type I interferon-mediated signaling pathway), GO:0060338 (regulation of type I interferon-mediated signaling pathway), GO:0060339 (negative regulation of type I interferon-mediated signaling pathway), GO:0060340 (positive regulation of type I interferon-mediated signaling pathway), GO:0071357 (cellular response to type I interferon).

NF-κB GO Terms Used for Targeted Gene Set Extraction: GO:0007252 (I-kappaB phosphorylation), GO:0033256 (I-kappaB/NF-kappaB complex), GO:0008384 (IkappaB kinase activity), GO:0008385 (IkappaB kinase complex), GO:0106137 (IkappaB kinase complex binding), GO:0051059 (NF-kappaB binding), GO:0071159 (NF-kappaB complex), GO:0035525 (NF-kappaB p50/p65 complex), GO:0007250 (activation of NF-kappaB-inducing kinase activity), GO:0007249 (canonical NF-kappaB signal transduction), GO:0032088 (negative regulation of NF-kappaB transcription factor activity), GO:0043124 (negative regulation of canonical NF-kappaB signal transduction), GO:1903721 (positive regulation of I-kappaB phosphorylation), GO:0051092 (positive regulation of NF-kappaB transcription factor activity), GO:0043123 (positive regulation of canonical NF-kappaB signal transduction), GO:1903719 (regulation of I-kappaB phosphorylation), GO:0043122 (regulation of canonical NF-kappaB signal transduction).
