# Supplementary material for: Optineurin Shapes Basal and LPS-Induced Transcriptomes in BV2 Microglia
Source: Int J Mol Sci. 2025 Oct 28;26(21):10453. doi: 10.3390/ijms262110453 (PMC12607547; doi:10.3390/ijms262110453)

Supplementary Figure 1. Optineurin expression and summary of unique and shared DEGs across BV2 conditions.

(A) Optineurin (Optn) transcript counts across experimental conditions. Data are shown as normalized expression values from RNA-Seq. (B) Summary table quantifying total, unique, and shared DEGs per comparison, with numbers and percentages of up- and downregulated genes. Differential expression was determined using DESeq2 (padj < 0.05, $|\log_2 FC| \geq 0.5$).

A

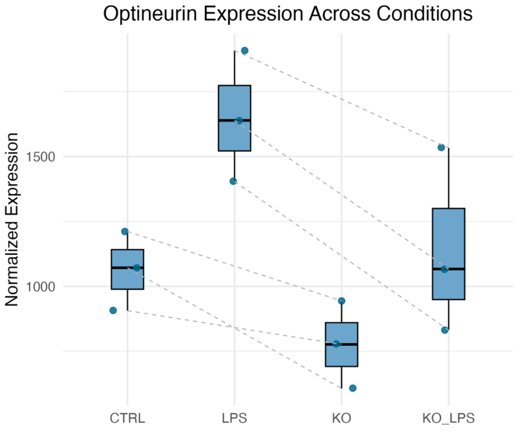

B

Comparison	Total Up	Unique_Up	Shared_Up	Total Down	Unique_Down	Shared_Down
**KO_vs_CTRL**	240 (22.5%)	45 (18.8%)	195 (81.2%)	825 (77.5%)	193 (23.4%)	632 (76.6%)
**LPS_vs_CTRL**	2726 (50.6%)	1471 (54.0%)	1255 (46.0%)	2661 (49.4%)	1793 (67.4%)	868 (32.6%)
**KO_LPS_vs_KO**	1502 (57.7%)	220 (14.6%)	1282 (85.4%)	1102 (42.3%)	188 (17.1%)	914 (82.9%)
**KO_LPS_vs_LPS**	1921 (47.3%)	1674 (87.1%)	247 (12.9%)	2138 (52.7%)	1437 (67.2%)	701 (32.8%)

Supplementary Figure 2. KEGG pathway enrichment analysis of differentially expressed genes in KO vs CTRL.

Dot plots show pathways enriched among significantly downregulated (left) and upregulated (right) genes in optineurin KO versus wild-type BV2 cells (padj < 0.05, $|\log_2 FC| \geq 0.5$).

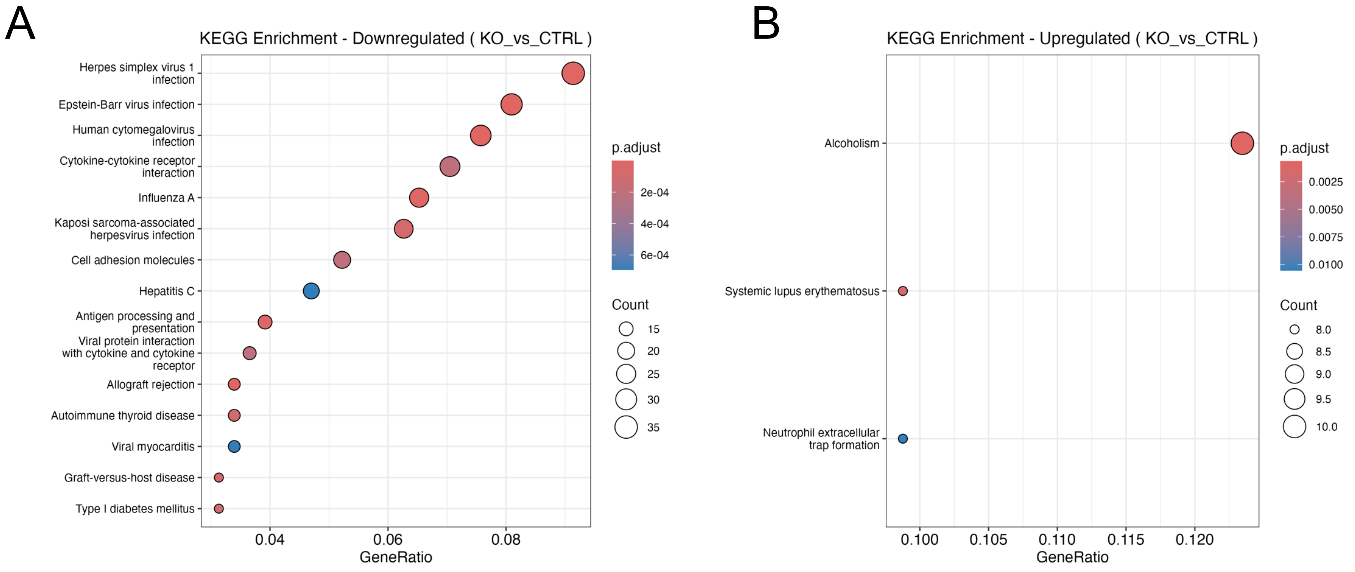

Supplementary Figure 3. GO enrichment of unique DEGs in LPS vs CTRL.

GO Biological Process enrichment analysis of unique DEGs identified in the LPS vs CTRL comparison (padj < 0.05, $|\log_2 FC| \geq 0.5$). Dot plots show enriched GO terms with dot size indicating the number of DEGs in the term (count), and color corresponding to adjusted p-value (padj). Top 10 up- and downregulated terms are shown.

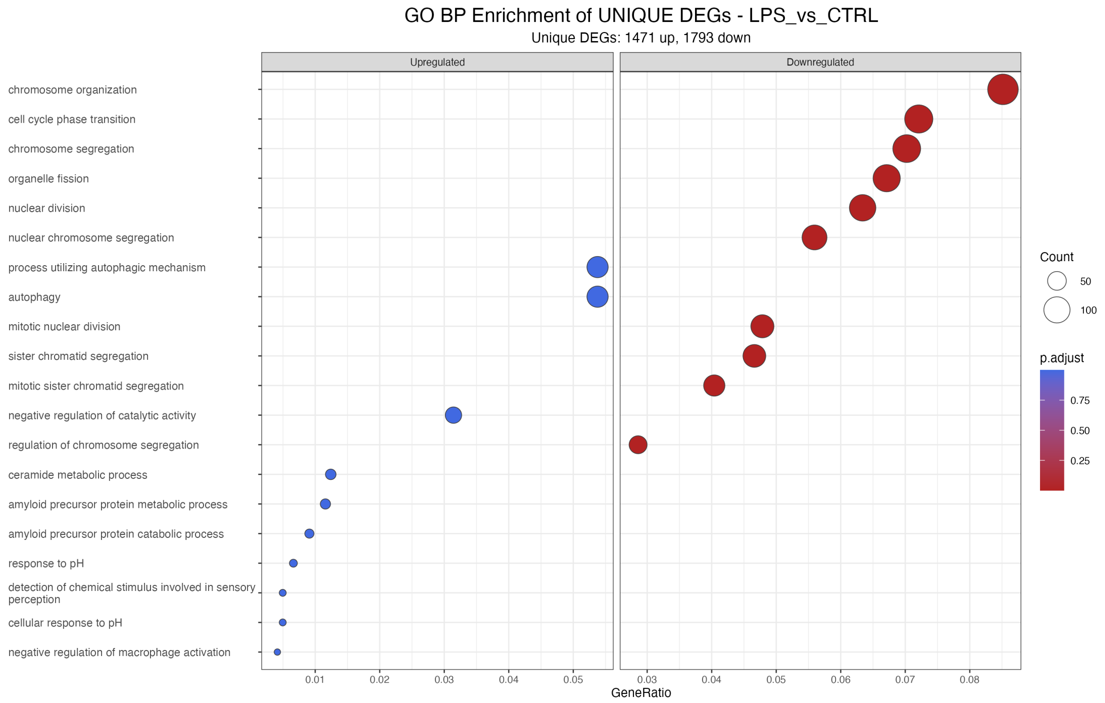

Supplementary Figure 4. GO enrichment of unique DEGs in KO_LPS vs KO.

GO Biological Process enrichment analysis of unique DEGs identified in the KO_LPS vs KO comparison (padj < 0.05, $|\log_2 FC| \geq 0.5$). Dot plots show enriched GO terms with dot size indicating the number of DEGs in the term (count), and color corresponding to adjusted p-value (padj). Top 10 up- and downregulated terms are shown.

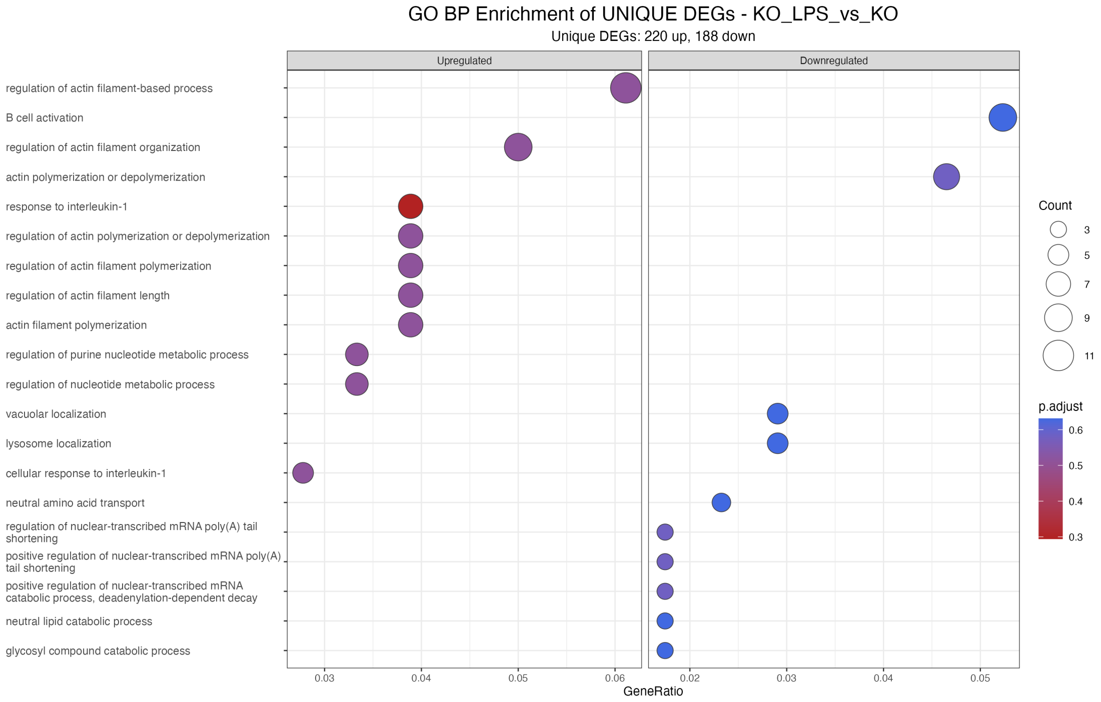

Supplementary Figure 5. Additional enrichment analyses for KO_LPS vs LPS.

(A) GO Biological Process enrichment of shared DEGs between LPS vs CTRL and KO_LPS vs LPS. (B) GO Biological Process enrichment of unique DEGs identified in KO_LPS vs LPS. (C) Reactome enrichment analysis of unique upregulated DEGs in KO_LPS vs LPS. (D–E) KEGG pathway enrichment analysis of all significantly downregulated (D) and upregulated (E) genes in KO_LPS vs LPS. Dot size indicates the number of DEGs in the term; color corresponds to adjusted p-value (padj).

A

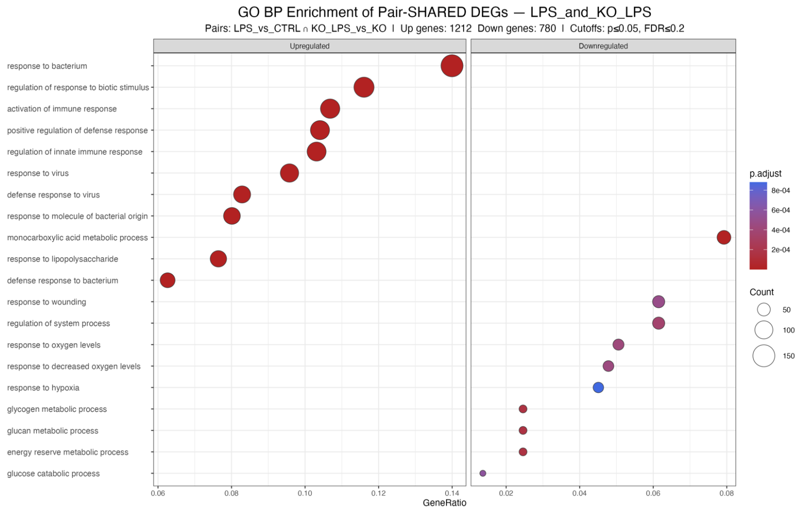

B

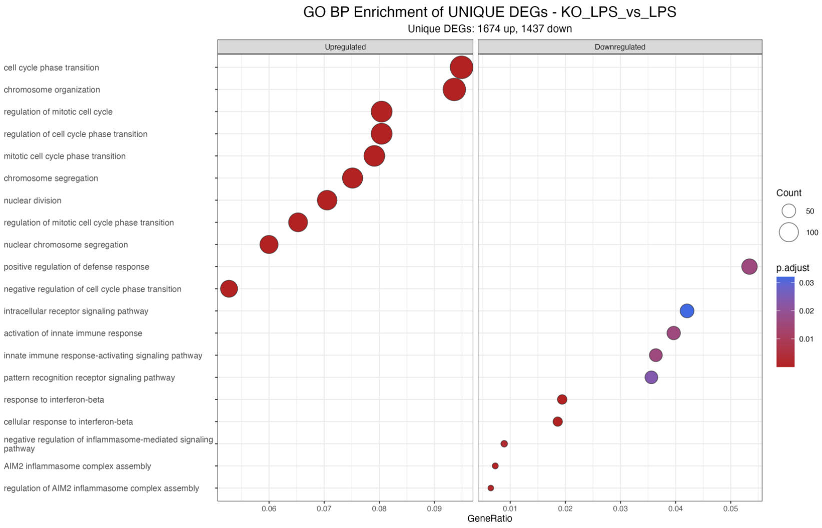

C

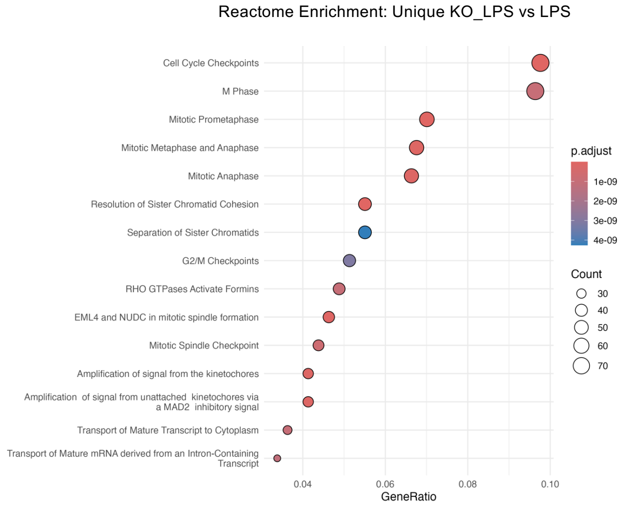

D

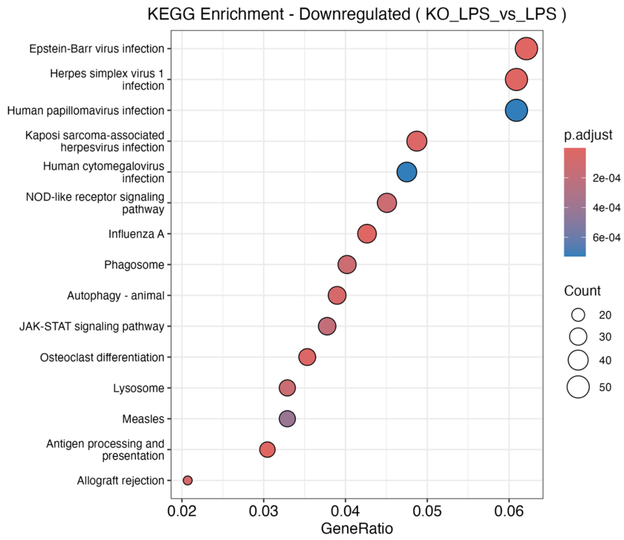

E

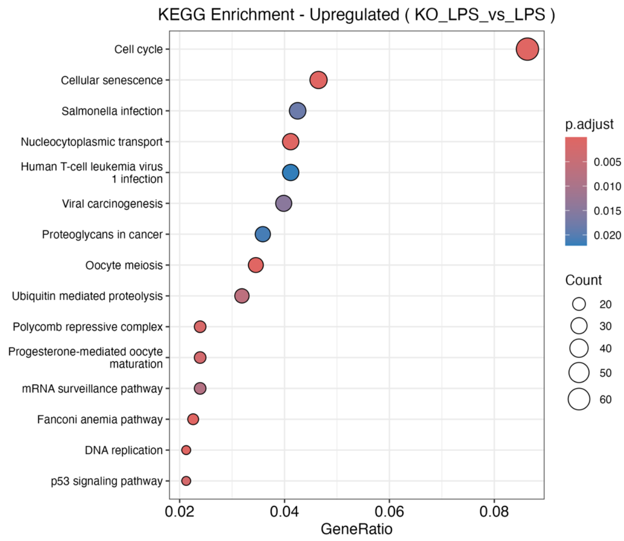

Supplementary Figure 6. Differential Regulation of Transcription Factors across key comparisons.

(A) Volcano plots of differentially expressed transcription factors (TFs) across three conditions: LPS vs CTRL (left), KO_LPS vs KO (middle), KO_LPS vs LPS (right) displaying $\log_2$ fold change versus $-\log_{10}$ adjusted p-value for all TFs (taken from AnimalTFDB), with significance thresholds of adjusted p-value < 0.05 and $|\log_2 FC| \geq 0.5$. The top 50 TFs by absolute $\log_2 FC$ are labeled. (B) Heatmaps showing z-scaled normalized expression (based on DESeq2 normalized counts) of the top 50 differentially expressed TFs for each comparison. Selection is based on combined ranking by adjusted p-value and absolute $\log_2$ fold change.

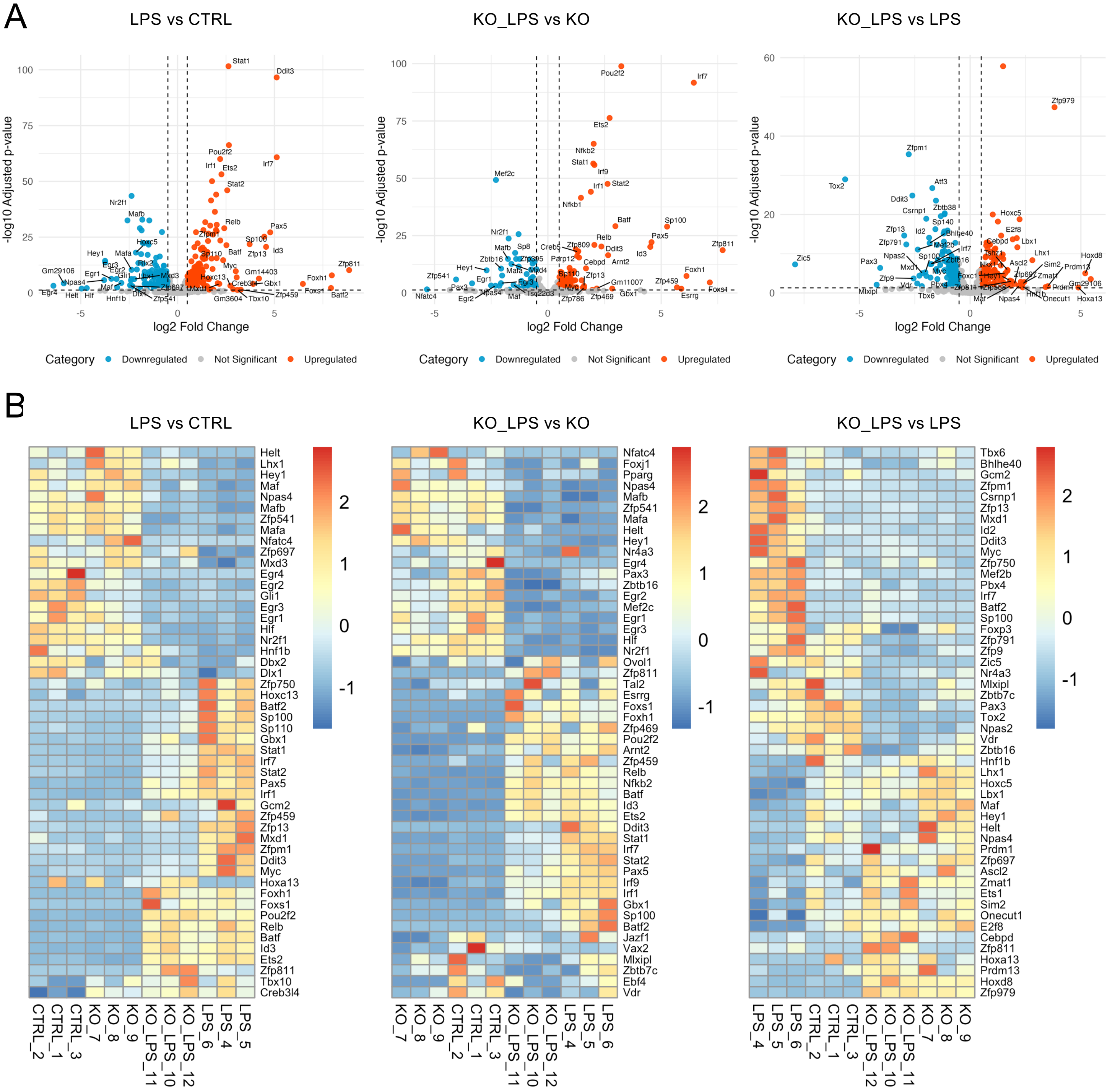

Supplementary Figure 7. Potential modulators of TBK1-optineurin-IRF3 axis.

A schematic representation of relationships among selected proteins and potential modulators was visualized by the SysWiz editor. Purple symbols indicate proteins/genes from the TBK1-optineurin-IRF3 axis and selected relevant DEGs from virus-, innate immunity-, and type I interferon-related pathways; remark: yellow hexagons indicate small molecule modulators; green hexagon indicates an approved drug, blue circles indicate recombinant peptide/protein modulators. Edges with arrowheads indicate activation/agonism, whereas edges with blunt bars indicate inhibition/antagonism. Proteins/modulators that could not be connected to any other molecule were omitted for clarity.

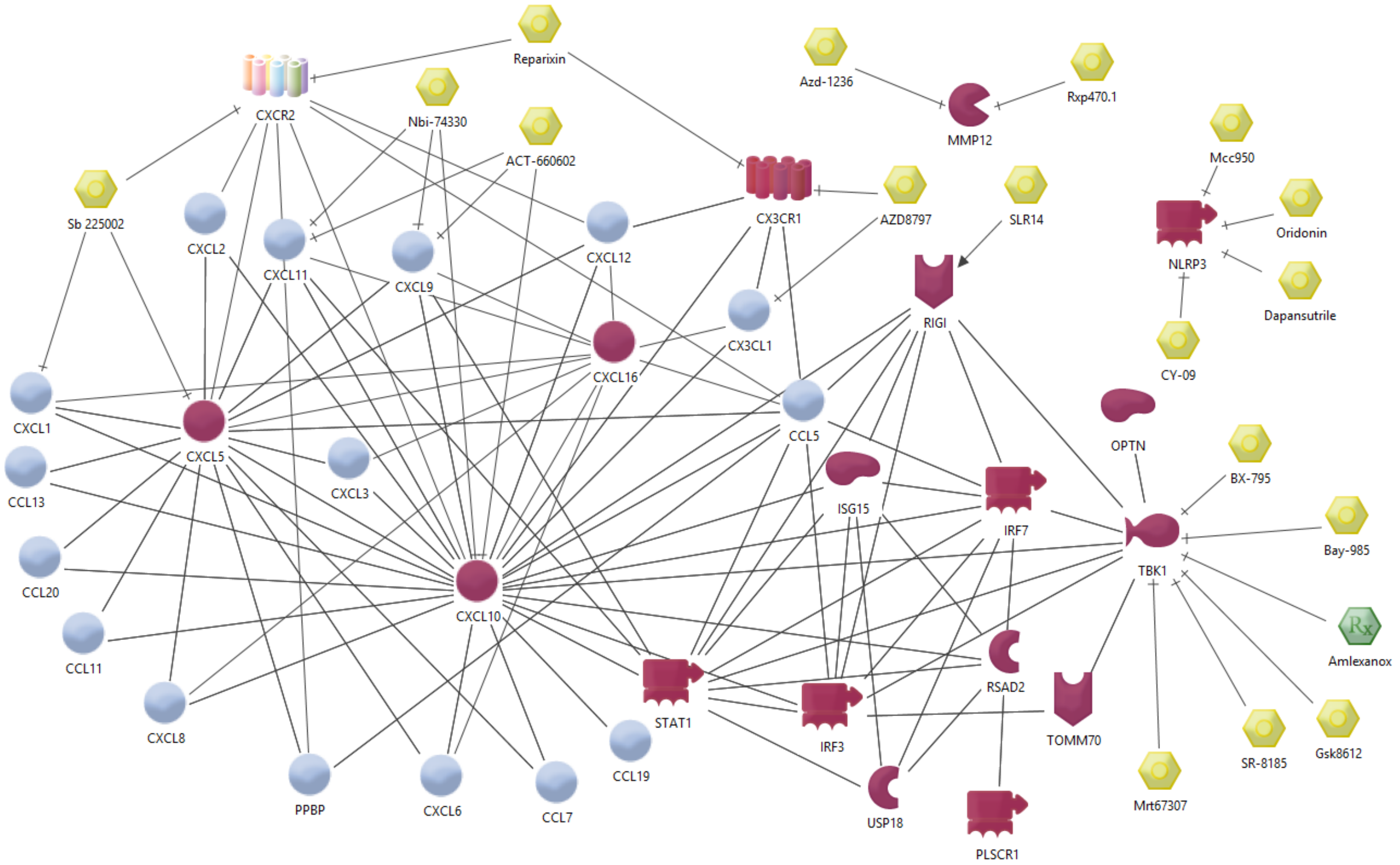

Supplement: Supplementary file 1 [file ijms-26-10453-s001.zip › ijms-3923307-supplementary-original.pdf]
